# Molecular detection, phylogenetic analysis and genetic diversity of recently isolated foot-and-mouth disease virus serotype A African topotype, Genotype IV

**DOI:** 10.1186/s12985-021-01693-y

**Published:** 2022-01-03

**Authors:** Ayah M. Hassan, Mostafa R. Zaher, Rabab T. Hassanien, Mervat I. Abd-El-Moniem, Ahmed R. Habashi, Essam M. Ibraheem, Momtaz A. Shahein, Mohamed E. El Zowalaty, Naglaa M. Hagag

**Affiliations:** 1 Genome Research Unit, Animal Health Research Institute, Agriculture Research Center (ARC), Dokki, Giza, 12618 Egypt; 2 Virology Research Department, Animal Health Research Institute, Agriculture Research Center (ARC), Dokki, Giza, 12618 Egypt; 3 Pathology Research Department, Animal Health Research Institute, Agriculture Research Center (ARC), Dokki, Giza, 12618 Egypt; 4 Zoonosis Science Center, Department of Medical Biochemistry and Microbiology, Uppsala University, SE-75 123 Uppsala, Sweden

**Keywords:** Foot and mouth disease virus, FMDV, Epidemiology, Variant, Genotype IV, Phylogenetic analysis, VP1 gene

## Abstract

**Background:**

Surveillance for circulating emerging diseases of economic importance has a major role in the rapid response to major pathogen outbreaks. Foot-and-mouth disease virus (FMDV) is one of the significant endemic viruses in Egypt. FMDV is periodically investigated for monitoring evolution and emergence of new variants. The genetic characterization of foot-and-mouth disease (FMD) virus serotype A responsible for recent outbreaks of FMD in Egypt was determined.

**Methods:**

Samples were collected from different locations and virus isolation was performed using BHK-21 cells. Viral RNA was extracted and samples were screened for FMDV using real-time RT-PCR. DNA sequence analysis was performed and computational and bioinformatics analyses were used to determine the substitution rates and phylogenetic relationship.

**Results:**

Sequence and phylogenetic analyses of full-length 1D region of FMDV samples collected from different governorates in 2020 showed close similarity to Egyptian FMDV strains from serotype A-African topotype-G-IV with genetic variation of 6.5%. Recently isolated FMDV strains showed high genetic variations from locally used vaccine strains in the major antigenic sites of VP1 region.

**Conclusions:**

Although, efforts made by the veterinary authorities to implement an effective mass vaccination plan, the recently detected FMDV strains in this study could not be subtyped using the FMDV primers routinely used for molecular serotyping. These dissimilarities raise the alarm for reconsideration of the FMDV isolates used in vaccine manufacture. Clearly close monitoring of FMD in Egypt is urgently required to define the risks of future outbreaks and to ensure appropriate control measures against FMD major outbreaks.

**Supplementary Information:**

The online version contains supplementary material available at 10.1186/s12985-021-01693-y.

## Introduction

Foot-and-mouth disease (FMD) is a highly contagious transboundary viral disease which affects large number of cloven-hoofed animals and causes severe economic losses due to reduction in milk yield and meat production, death of young animals, costs of medication, and restriction of animal movement from endemic countries [1–3].

Foot-and-mouth disease virus (FMDV) is a member of the genus *Aphthovirus* in the family *Picornaviridae*. FMDV is a small, non-enveloped, positive-sense, single stranded, non-segmented RNA virus surrounded by an icosahedral capsid with approximately 60 copies of four structural viral proteins (VP1, VP2, VP3, and VP4) [4]. FMDV has seven immunologically distinct serotypes A, O, C, Sothern African territories (SAT) 1, SAT 2, SAT 3 and Asia. Within these serotypes, more than 65 diversities of topotypes, genetic lineages, and strains have also been identified [4, 5].

The replication process of FMD viral RNA is prone to high level of replication errors which is responsible for high level of genetic diversity between virus serotypes which share around 86% homology [6, 7]. VP1 is a highly variable protein which contains the major immunogenic epitopes. It has 30–50% inconsistency between the seven serotypes. This variable protein plays an important role in the molecular epidemiology investigations of FMDV globally [8]. Moreover, phylogenetic analyses through multiple alignments nucleotide sequences of VP1 help determine the variation, genetic relationship and geographical circulation among different FMDV serotypes [9, 10]. VP1 also contains serotype-specific amino acids which enable the differentiation between different serotypes [11].

The epidemiology of FMD in Egypt is complicated because of the circulation of endemic FMD viruses as well as incursions of exotic viral strains from the Middle East and Sub-Saharan Africa [12]. Egypt has been considered one of the FMDV endemic African countries with multiple outbreaks since 1958 [13]. FMDV serotypes O, A and SAT2 strains are co-circulating in Egypt. There were several topotypes of serotype A which have been reported in the country according to the reports of the World Reference Laboratory for Foot and Mouth Disease (WRLFMD, Pirbright, UK). In 2006, serotype A caused severe outbreaks and there was a homology between the Egyptian strains and East African strains [14]. An Asian topotype which resembles the Iranian strain (A-Iran-05^BAR-08^) was introduced in Egypt for first time during 2010, and the last laboratory report was during 2015 [15, 16]. On the other hand, FMDV serotype A topotype Africa G-IV strain was recorded for the first time during 2012 and then was reported in some occasions during 2016 and 2018 [15, 16].

Because of the high mutation rate of FMDV and the possibility of new FMDV strains to emerge, there is a crucial requirement for continuous monitoring, genetic and antigenic characterization of the circulating FMDV strains. The surveillance for newly emerging strains will allow a rapid response to avoid its spread and ensure effective vaccination programs. Identification of new FMDV variants will help include them into vaccine production pipelines to maintain appropriate and updated control measures. Thus, the aim of the current study was to determine the molecular and genetic characterization of a new FMDV serotype A circulating variant in Egypt during 2020 through complete sequencing of VP1 gene.

## Materials and methods

### Sample collection

Clinical samples were collected from 28 animals (26 epithelium and two vesicular fluid samples); each sample in the study is a representative for each animal. (S1-Additional file 1).

Criteria for selection of suspected animals depended mainly on the appearance of FMD clinical symptoms (salivation, tongue and mouth vesicles appearance, depression, and loss of appetite). Epithelial tissue was collected from an unruptured or recently ruptured vesicle, from the tongue or buccal mucosa, and was placed in a viral transport medium with added antibiotics. Vesicular fluid was withdrawn from the unruptured vesicle using a sterile needle and was submitted in sterile sample container. All collected samples were submitted in ice container containing ice packs to maintain cold chain temperature during transport of the samples to the laboratory. The samples were collected between October and December 2020 from clinically diagnosed cattle and buffaloes (showing symptoms suspected to be FMD) from five governorates in Egypt; Behera (n = two), Cairo (n = seven), Minya (n = five), Port Said (n = six) and Qalyubia (n = eight). The clinical disease was reported in both vaccinated and non-vaccinated animals however, diseased animals in Port Said and Qalyubia were vaccinated with a local polyvalent inactivated vaccine manufactured by the Veterinary Serum and Vaccine Research Institute (VSVRI, Cairo, Egypt).

### Virus isolation

Baby Hamster Kidney (BHK-21) cells were used for FMD virus isolation as was previously described [17]. Approximately, cells of ninety percentage of confluent monolayer were prepared in 25 cm^3^ cell culture flasks containing minimum essential medium (MEM) with Earl's salts supplemented with 10% fetal calf serum, 100 IU/ml penicillin, and 100 mg/ml streptomycin. The growth media supernatant was decanted from the flask and then the cell monolayer was washed three times with sterile phosphate buffer saline and 0.5 ml of the prepared viral sample was inoculated gently onto the cell monolayer. Flasks were incubated for 1 h at 37°C in the presence of 5% CO_2_. This was followed by the addition of MEM and cells were incubated for 24-72 hr at 37°C in the presence of 5% CO_2_. Inoculated cells were harvested when cytopathic effects (CPE) were observed and cells were frozen and thawed three times and were passaged for three successive blind passages in the same way.

### Viral RNA extraction

Viral RNA was extracted using EasyPure viral RNA kit (TransGen Biotech, Beijing, China) according to the manufacturer's instructions. Briefly, FMDV RNA was isolated from 200 μL sample as starting materials and RNA was eluted in a final volume of 30 µl RNase-free water. RNA extracts were either used directly after extraction or kept at − 20 °C for further analysis.

### Real-time RT-PCR (qRT-PCR) and one-step RT-PCR

Extracted samples were screened for the presence of FMDV RNA using primers and probe directed toward the conserved 3D gene (Table 1). TransScript® Probe One-Step qRT-PCR SuperMix (TransGen, Beijing, China) was used according to  the manufacture's instruction for the amplification of 3D gene. The PCR cycles consisted of 45 °C for 5 min of reverse transcription, then initial denaturation at 94 °C for 2 min, 40 cycles of 94 °C for 5 s and 60 °C for 30 s with fluorescence data collection in this step each cycle. Samples with cycle threshold (C_t_) lower than 30 were used for the conventional one-step RT-PCR. The complete VP1 gene was amplified using the EasyScript One-Step RT-PCR SuperMix (TransGen, Beijing, China). Briefly, a total of 5 μL of extracted viral RNA was used in the RT-PCR which consisted of 12.5 μL of reaction mix, 0.4 μL of enzyme mix, and 3.1 μL of RNase-free water were mixed with 2 μL of each primer (10 μM) into a final volume of 25 µl. One-Step RT-PCR was performed using the following conditions: 45 °C for 25 min. (reverse transcription step), 94 °C for 5 min, followed by 40 cycles of 94 °C for 45 s and 60 °C for 1 min. except for SAT2 specific detection primer at 50 °C for 45 s. and 72 °C for 45 s followed by a final extension at 72 °C for 10 min. The primers targeting the variable region in the 1D gene of viral RNA are shown in Table 1.

**Table 1:** Primers used in real-time RT-PCR of 3D gene, one-step RT-PCR and sequencing reaction of 1D gene of FMDV

Serotype	Primer designation	Primer sequence (5'-3')	Amplicon size	References
All serotypes	3D-F	ACTGGGTTTTACAAACCTGTGA	106 bp	[20]
	3D-R	GCGAGTCCTGCCACGGA		
	3D-Probe	TCCTTTGCACGCCGTGGGAC		
O	O-Egy-F	CCTCCTTCAAYTACGGT	283 bp	[21]
A	A–1C612F	TAGCGCCGGCAAAGACTTTGA	814 bp	[14]
SAT2	SAT2-Egy-F	TGAYCGCAGTACACAYGTYC	666 bp	[22]
A,O, and SAT2	EUR–2B52R	GACATGTCCTCCTGCATCTGGTTGAT	–	[14]
All serotypes (pan-FMDV)	FMD-3161-F	TCG CVC AGT ACT ACR CAC AGT A	1143 bp	[23]
	FMD-4303-R	TGA CGT CRG AGA AGA AGA ARG G		

Following the amplification of RT-PCR products in the programmable thermal cycler T100 (BioRad, USA), the PCR products were analyzed on 1.2% agarose gel electrophoresis system using Tris Borate EDTA buffer (1X) stained with ethidium bromide. A 5 μL of the RT-PCR products, as well as the DNA ladder, were loaded into the preformed wells. DNA fragments of positive samples were excised from the agarose gel and the amplified RT-PCR products were purified from the gel using QIAquick Gel Extraction Kit (Qiagen, USA) according to the manufacturer’s instructions and DNA was eluted in a final volume of 30 µl. Determination of DNA concentration was performed using Qubit™ dsDNA HS assay kit (Molecular Probes, Life technologies, USA) using the Qubit® 2.0 fluorometer for accurate DNA quantification.

### DNA sequencing

The purified PCR products were sequenced in both directions using the dideoxy chain termination method using the same primers (Table 1). Sequencing reaction was prepared using BigDye® Terminator v3.1 Cycle Sequencing Kit (Thermo Fisher, USA) with 3.2 picomol concentrations of both the forward and reverse primers for each positive sample in two different reactions.

Thermal cycling conditions for sequencing were at 96 °C for 1 min, followed by 25 cycles at 96 °C for 10 s. 50 °C for 5 s and 60 °C for 2 min using T-100TM Thermal Cycler (Bio-Rad, USA). Sequencing reaction product was purified with Centri-Sep™ Spin Columns (Thermo Fisher, USA), followed by electrokinetic injection on capillary electrophoresis systems 3500 Genetic analyzer (Applied Biosystems, USA).

### Phylogenetic analysis

Computational and bioinformatics analyses were used to determine the substitution rates and construct a phylogenetic tree [18, 19]. Nucleic acid and amino acid sequence similarities were determined using the Basic Local Alignment Search Tool (BLAST) (https://blast.ncbi.nlm.nih.gov/Blast.cgi) available at NCBI using default parameters. Results produced from local alignment were used to determine the sequence to be used in multiple alignment analysis. Multiple sequence alignment (MSA) was conducted using ClustalW/BioEdit software—version 7.1 [24]. Results of MSA were trimmed and stripped from columns containing gaps and phylogenetic tree was generated using the neighbor-joining method with bootstrapping over 1000 replicates using MEGA software version X [19, 25]

## Results

### FMDV screening using qRT-PCR

The 28 collected samples were initially screened for FMDV using qRT-PCR and 22 (78.5%) samples were positive using pan-FMDV 3D primers set [20]. The C_t_ values of positive samples were in the range of 21 to 28.

### Virus isolation

Virus culture was performed for six of the FMDV positive samples (four epithelial and two vesicular samples) and the samples were selected according to their C_t_ values. The cytopathic effects (CPE) of FMDV on BHK-21 cells were observed in the form of rounding, swelling, clumping, or sloughing of the cells or monolayer detachment from the surface of the cell culture flasks. After 24–72 h of inoculation, cells were severely damaged and finally cell death occurred which indicated the presence of infectious virus. Nevertheless, samples that did not show any CPE didn't not induce any morphologic changes to BHK-21 cells.

### Sequencing and genetic characterization

FMDV positive samples by qRT-PCR were further tested using serotypes A, O, and SAT2 specific primers, and surprisingly samples could not be identified using the previously reported serotype-specific primers (Table 1).

Further analysis was performed using FMD-3161-F/ FMD-4303-R primers which have high sensitivity for all FMDV serotypes [23]. These primers were designed to anneal within the VP3 coding region (forward primer) and the 2B coding region (reverse primer) to amplify the full length VP1 coding region. All the 22 pan-FMDV positive isolates were found to be positive for serotype A using conventional RT-PCR.

Full-length 1D (VP1 encoding) gene sequences of six FMDV isolates generated in the present study were deposited in GenBank under the accession numbers; MW413345, MW413346, MW413347, MW413348, MW413350, and MW413351. Additional short sequence was also generated and was deposited in GenBank under accession number MW413349. BLAST analysis of the recently detected and circulating Egyptian FMDV strains in the present study showed a close relationship with sequences of Egyptian FMD viruses isolated between late 2015 and early 2016 with high nucleotide similarity ranged from 92.7 to 94.76% with FMDV/A/buffalo/Damietta/Egypt/2016 (accession number MT863268) and FMDV/A/Ismailia 1/Egy/2016 (accession number KX446996), respectively.

Sequences of recently isolated samples in the present study and sequences of BLAST first 10 hits as shown in Table 2 were used to construct an identity matrix using MegAlign Pro, DNA-STAR software (Additional file 2). Sequences of the six strains showed similarity within each other with a mean value of 98.4%.

**Table 2:** Pairwise results of recent Egyptian FMDV isolate (A/Egy/1028) showing first 10 Hits of BLAST and the most closely related prototype and result of alignment with local vaccinal strains

No	Description	Accession number	Per. query coverage (%)	Per. identity (%)	Serotype	Topotype	Genotype	Remarks
1	Ismailia 1/Egy/2016	KX446996.1	100	94.79	A	Africa	IV	First 10 Hits of BLAST result
2	A/EGY/8/2015	MK422573.1	100	94.47	A	Africa	IV	
3	Ismailia 2/Egy/2016	KX446997.1	100	94.47	A	Africa	IV	
4	A/Egy/Faiyum/2016	MG552843.1	100	94.31	A	Africa	IV	
5	A/cow/Dakahlia/Egypt/2016	MT863264.1	100	94.31	A	Africa	IV	
6	A/buffalo/Damietta/Egypt/2016	MT863268.1	100	94.15	A	Africa	IV	
7	A/buffalo/Damietta/Egypt/2016	MT863266.1	100	94.15	A	Africa	IV	
8	ETH/19/2015 VP1 gene	MN987497.1	100	93.84	A	Africa	IV	
9	Beni-suef1/Egy/2017	MF322693.1	100	93.84	A	Africa	IV	
10	A/SUD/6/2018	MK422591.1	100	93.05	A	Africa	IV	
11	SUD/3/77	GU566064.1	100	84.25	A	Africa	IV	Prototype sequence
12	EGY 1/2012	KC440882.1	88	78.25	A	ASIA	Iran-05	Local vaccine strain

### Phylogenetic analysis

**Fig. 1:**
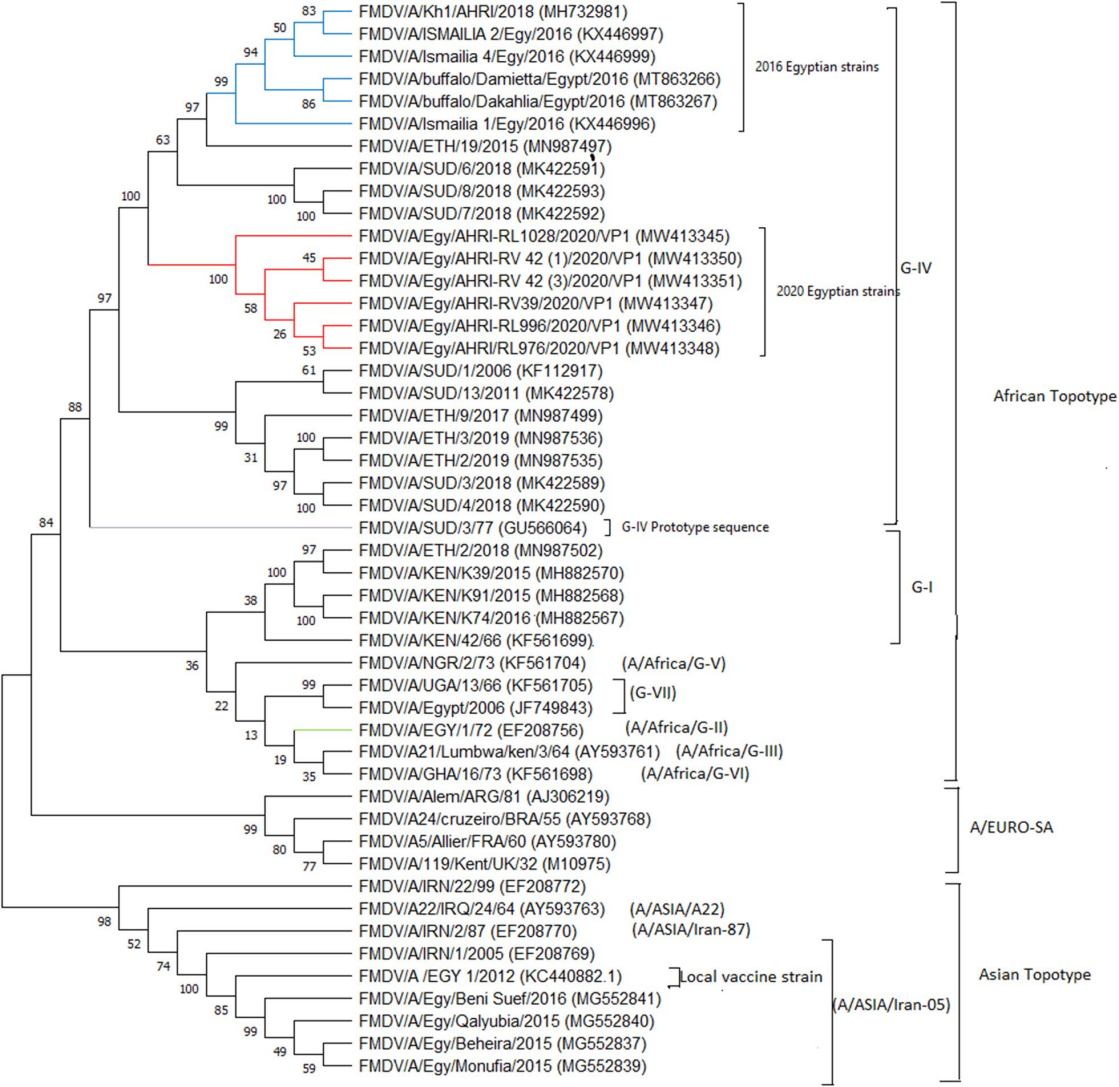
Phylogenetic tree using the neighbor-joining method based on VP1 gene (636 nucleotides) of the six FMDV isolates sequences generated in the present study with additional 42 sequences of FMDV serotype A retrieved from the GenBank (https://www.ncbi.nlm.nih.gov/genbank) database. Numbers at the internal nodes represent the bootstrap probabilities (1000 replicates)

Multiple sequence alignment and phylogenetic analyses showed that the recently circulated FMDV strains grouped in a separate cluster (Fig. 1). This may be explained due to the close similarity among the recently circulating strains and their variation from the previously reported Egyptian FMDV strains of the same genotype. Although the phylogenetic tree included the prototype sequence of all the African genotypes and some of the Asian and Euro-South American genotypes, the newly reported sequences in the current study showed a common ancestor with prototype sequence (A/Africa/Sud/77), the reference sequence of African genotype-IV, confirming their relatedness to the later genotype.

## Discussion

Whilst Egypt is undertaking an extensive FMD control program through vaccinating animals with a local inactivated polyvalent vaccine consisting of serotype O pan Asian II (EGY/2010), A Iran 05 (A/EGY/1/2012), SAT2 (EGY/Gharbia/2012) and SAT2 (LIB/2018), FMD remains an endemic disease in the country. The vaccination program was successful in reducing the incidence of FMD; however, FMDV strains remain circulating due to the uncontrolled animal movement. Moreover, the unique geographical location of Egypt poses a considerable risk for the incursion of circulating strains from Asian and East African countries. During the past decade, there were at least eight strains of FMDV serotypes (A, O, and SAT2). These strains included (A-Iran 05, A-African genotype IV, A-African genotype VII, O-Pan Asia II, O East Africa, SAT2 Ghb-12, SAT2 Lib-12, and SAT2 Alx-12) with alternative displacement and winter peak of 2 to 3 circulating strains and a dominant circulating strain every year [26].

In the present study, all sequenced FMDV isolates were genetically characterized as serotype A of genotype IV of the African topotype, as indicated by the first 10 hits of BLAST results which belongs to this lineage [27, 28].

The genotype of the new strains was confirmed to be G-IV by the significant similarity of 84.2% to the reference prototype sequence A/Africa/SUD/77 (accession number GU566064).  The generated FMDV sequences in the present study also showed similarity to previously reported sequences from Sudan (93.2%–85.6%) and Ethiopia (94%–83.7%), respectively as shown in Table 2. These results suggested the possibility of genotype-IV introduction to the Egyptian territories directly from Ethiopia or Sudan as was previously reported [29].

**Fig. 2:**
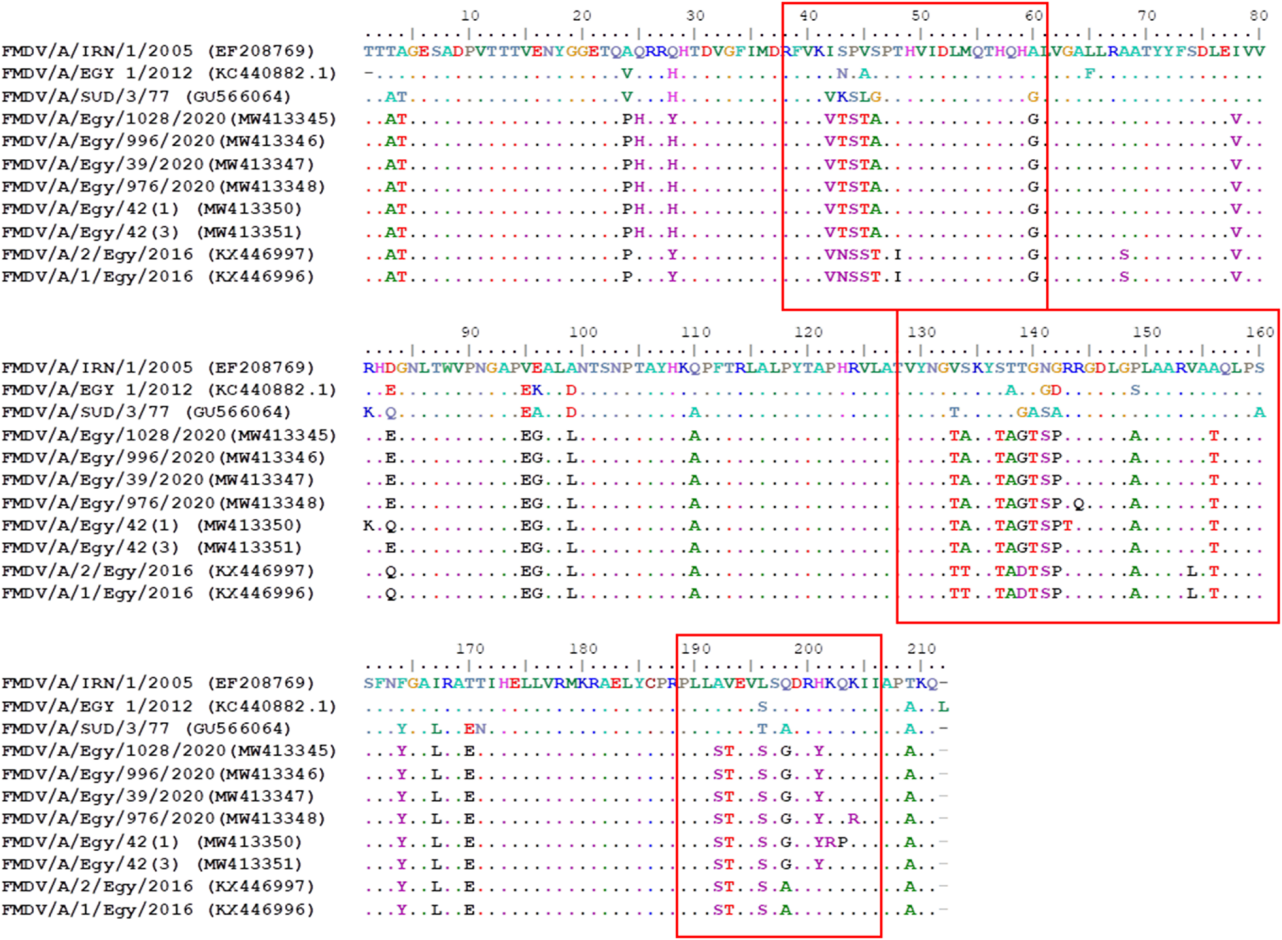
Deduced amino acid sequence alignment of VP1 of the new FMDV isolates in the present study compared with other FMDV/G-IV isolates. The red squares denote the major variable sites chosen in the comparison.

As shown from the results of local alignment and phylogenetic analyses, the sequences of the current circulating FMDV serotype A isolates are deviating from the previously reported Egyptian sequences either African genotype-VII or Asian genotype-Iran-05. Similarly, the sequence variability of these strains was found considerably high when they were aligned with the local vaccine strains A/IRN/1/2005 (accession number EF208769) and A/EGY/1/2012 (acession number KC440882) (Table 3 and Additional file 2). Further investigation of the major antigenic regions of VP1 showed the occurence of multiple amino acid substitutions in the recently circulating strains as compared to the vaccinal strains used in the local FMD vaccines in Egypt (Fig. 2).

**Table 3:** Amino acid variations in the major antigenic sites of VP1 between the recently circulating FMDV strains and the vaccinal strains in Egypt

No	Residue position	Viral strain		
		Vaccine strain (A/Iran-05)	Vaccine strain (A/Egy/1/2012)	New Egyptian samples
1	42	I	I	V
2	43	S	N	T
3	44	P	P	S
4	45	V	A	T
5	46	S	S	A
6	60	A	A	G
7	133	V	V	T
8	134	S	S	A
9	137	S	S	T
10	138	T	A	A
11	139	T	T	G
12	140	G	G	T
13	141	N	G	S
14	142	G	D	P
15	149	P	S	A
16	156	A	A	T
17	198	Q	Q	G
18	201	H	H	Y

It has also been reported that the vaccine strain of the origin (Iran-05) showed no protection against G-IV strains by vaccine matching challenge test as was previously reported by the WRLFMD [30].

Comparison of amino acid constitution between the new strains and vaccinal strains A/IRN/1/2005 and A/EGY/1/2012 showed major amino acid variation in the FMDV antigenic sites. Those regions span from (40–60), (131–160) and (195–202) of VP1 amino acids. Most of the amino acid substitutions in those sites were conservative. However, there were some residues that were identified as surface or major antigenic sites [31], which are non-conservative substitutions and may affect the vaccine efficiency towards the current circulating strains. Such changes may show difference in the exposed residue polarity such as V60T in (A/EGY/1/2012) and G141S in (A/EGY/1/2012). Others caused non-conservative change in amino acid structure such as P149A in (A/Asia/Iran-05).

## Conclusion

The findings of the present study demonstarte that the recently circulating FMDV strains in Egypt in 2020 belong to serotype A, Africa topotype G-IV, and the detected strains showed a significant genetic variation from the previously reported strains in Egypt with a mean value of 93.7%. To the authors' knowldege, strain A Africa G-IV is not included in the currently deployed local vaccines in Egypt which may result in risk of future FMD outbreaks and may increase the chance of further genetic mutations. Eventually, the findings of the present study highly recommend the reevaluation of the FMD vaccines currently used in Egypt to avoid the recurrence of FMDV outbreaks.

## Supplementary Information

**Additional file 1. Table (1):** Samples collected in the present and the selected FMDV positive samples used for VP1 sequencing and their accession numbers.

**Additional file 2.** The percentage of nucleotides identity and divergence between FMDV strains in the present study and the published reference strains retrieved from GenBank based on the complete sequence of VP1.

## Data Availability

Sequence data generated in the present study that support the findings of this study are publically available and were deposited in GenBank/NCBI/NLM under accession numbers MW413345, MW413346, MW413347, MW413348, MW413349, MW413350, and MW413351.

## Abbreviations

CPE: Cytopathic effects
FMDV: Foot-and-mouth disease virus
FMD: Foot-and-mouth disease
RT-PCR: Reverse-transcription polymerase chain reaction
qRT-PCR: Real time reverse-transcription polymerase chain reaction
