# Supplementary material for: Molecular detection, phylogenetic analysis and genetic diversity of recently isolated foot-and-mouth disease virus serotype A African topotype, Genotype IV
Source: Virol J. 2022 Jan 3;19:1. doi: 10.1186/s12985-021-01693-y (PMC8722054; doi:10.1186/s12985-021-01693-y)
Supplement: Supplementary file 1 — Additional file 1. Table (1): Samples collected in the present and the selected FMDV positive samples used for VP1 sequencing and their accession numbers. [file 12985_2021_1693_MOESM1_ESM.docx]

**Molecular detection, phylogenetic analysis and genetic diversity of recently isolated foot-and-mouth disease virus serotype A African topotype genotype IV**

Ayah M. Hassan,^1^ Mostafa R. Zaher,^1^ Rabab T. Hassanien,^2^ Mervat I. Abd-El-Moniem,^2^ Ahmed R. Habashi,^2^ Essam M. Ibraheem,^3^ Momtaz A. Shahein,^2^ Mohamed E. El Zowalaty,^4 ,*^ Naglaa M. Hagag^1,*^

^1^ Genome Research Unit, Animal Health Research Institute, Agriculture Research Center (ARC), 12618 Dokki, Giza, Egypt

^2^ Virology Research Department, Animal Health Research Institute, Agriculture Research Center (ARC), 12618 Dokki, Giza, Egypt

^3^Pathology Research Department, Animal Health Research Institute, Agriculture Research Center (ARC), 12618 Dokki, Giza, Egypt

^4^ Department of Medical Biochemistry and Microbiology, Zoonosis Science Center, Uppsala University, SE-75 123 Uppsala, Sweden

Corresponding Authors: MEZ ([elzow005@gmail.com](mailto:elzow005@gmail.com)) and NMH ([naglaahagagahri@gmail.com](mailto:naglaahagagahri@gmail.com))

Table (1): Samples collected in the present and the selected FMDV positive samples used for VP1 sequencing and their accession numbers.

| **Accession number** | **Molecular Tests results** | **Virus Isolation results** | **Vaccination** | **Sample type** | **Species** | **Sample number** | **Location** | **Date of collection** | **Sample Code** |
| --- | --- | --- | --- | --- | --- | --- | --- | --- | --- |
| MW413347 | Positive | Yes | Un known | Epithelium | Cattle | 1 | Behera | 5/10/2020 | RV/39 |
| --- | Positive | No | Unknown | Epithelium | Cattle | 2 |  |  |  |
| --- | Positive | No | Local vaccine | Epithelium | Cattle | 1 | Port Said | 11/10/2020 | RV/40 |
| --- | Positive | No | Local vaccine | Epithelium | Buffalo | 2 |  |  |  |
| --- | Positive | No | Local vaccine | Epithelium | Buffalo | 3 |  |  |  |
| --- | Positive | No | Local vaccine | Epithelium | Buffalo | 4 |  |  |  |
| MW413348 | Positive | Yes | Local vaccine | Vesicular fluid | Cattle | 1 | Port Said | 18/11/2020 | RL/976 |
| --- | Positive | No | Local vaccine | Epithelium | Cattle | 2 |  |  |  |
| MW413346 | Positive | Yes | Un known | Epithelium | Cattle | 1 | Minya | 2/11/2020 | RL/996 |
| --- | Positive | No | Un Known | Epithelium | Cattle | 2 |  |  |  |
| --- | Positive | No | Un Known | Epithelium | Cattle | 1 | Minya | 8/11/2020 | RL/1007 |
| --- | Positive | No | Un Known | Epithelium | Cattle | 2 |  |  |  |
| --- | Positive | No | Un Known | Epithelium | Cattle | 3 |  |  |  |
| MW413345 | Positive | Yes | Local vaccine | Epithelium | Cattle | 1 | Qalyubia | 18/11/2020 | RL/1028 |
| --- | Positive | No | Local vaccine | Epithelium | Cattle | 2 |  |  |  |
| --- | Negative | No | Local vaccine | Epithelium | Cattle | 1 | Qalyubia | 19/11/2020 | RV/41 |
| --- | Negative | No | Local vaccine | Epithelium | Cattle | 2 |  |  |  |
| --- | Negative | No | Local vaccine | Epithelium | Buffalo | 3 |  |  |  |
| --- | Negative | No | Local vaccine | Epithelium | Cattle | 4 |  |  |  |
| --- | Negative | No | Local vaccine | Epithelium | Cattle | 5 |  |  |  |
| --- | Negative | No | Local vaccine | Epithelium | Cattle | 6 |  |  |  |
| MW413350 | Positive | Yes | Un Known | Vesicular fluid | Cattle | 1 | Cairo | 17/12/2020 | RV/42 |
| --- | Positive | No | Un Known | Epithelium | Buffalo | 2 |  |  |  |
| MW413351 | Positive | Yes | Un Known | Epithelium | Cattle | 3 |  |  |  |
| --- | Positive | No | Un Known | Epithelium | Cattle | 4 |  |  |  |
| --- | Positive | No | Un Known | Epithelium | Cattle | 5 |  |  |  |
| --- | Positive | No | Un Known | Epithelium | Cattle | 6 |  |  |  |
| --- | Positive | No | Un Known | Epithelium | Cattle | 7 |  |  |  |
