# Supplementary material for: Molecular detection, phylogenetic analysis and genetic diversity of recently isolated foot-and-mouth disease virus serotype A African topotype, Genotype IV
Source: Virol J. 2022 Jan 3;19:1. doi: 10.1186/s12985-021-01693-y (PMC8722054; doi:10.1186/s12985-021-01693-y)
Supplement: Supplementary file 2 — Additional file 2. The percentage of nucleotides identity and divergence between FMDV strains in the present study and the published reference strains retrieved from GenBank based on the complete sequence of VP1. [file 12985_2021_1693_MOESM2_ESM.docx]

**Molecular detection, phylogenetic analysis and genetic diversity of recently isolated foot-and-mouth disease virus serotype A African topotype Genotype IV**

Ayah M. Hassan,^1^ Mostafa R. Zaher,^1^ Rabab T. Hassanien,^2^ Mervat I. Abd-El-Moniem,^2^ Ahmed R. Habashi,^2^ Essam M. Ibraheem,^3^ Momtaz A. Shahein,^2^ Mohamed E. El Zowalaty,^4 ,*^ Naglaa M. Hagag^1,*^

^1^ Genome Research Unit, Animal Health Research Institute, Agriculture Research Center (ARC), 12618 Dokki, Giza, Egypt

^2^ Virology Research Department, Animal Health Research Institute, Agriculture Research Center (ARC), 12618 Dokki, Giza, Egypt

^3^Pathology Research Department, Animal Health Research Institute, Agriculture Research Center (ARC), 12618 Dokki, Giza, Egypt

^4^ Department of Medical Biochemistry and Microbiology, Zoonosis Science Center, Uppsala University, SE-75 123 Uppsala, Sweden

Corresponding Authors: MEZ ([elzow005@gmail.com](mailto:elzow005@gmail.com)) and NMH ([naglaahagagahri@gmail.com](mailto:naglaahagagahri@gmail.com))

Additional file 2:


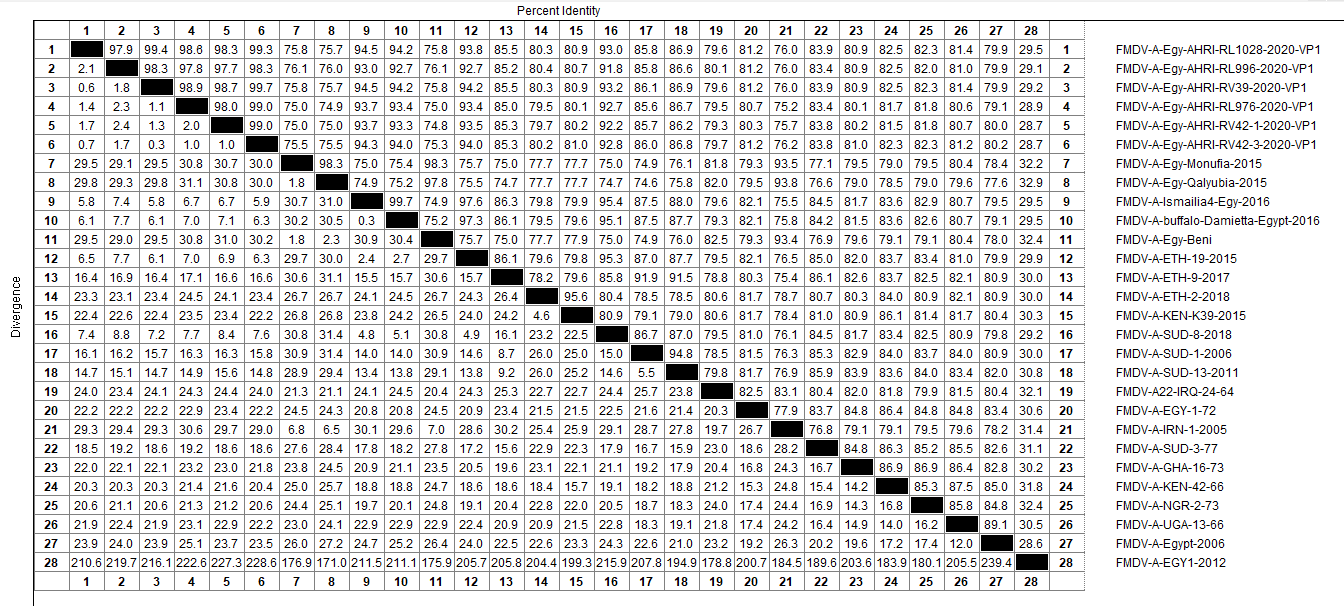


Percentage identity and divergence between FMDV strains in the present study and the published reference strains retrieved from GenBank based on the complete nucleotide sequence of VP1.
